# Association Analysis between the Polymorphisms of *HSD11B1* and *H6PD* and Risk of Polycystic Ovary Syndrome in Chinese Population

**DOI:** 10.1371/journal.pone.0140326

**Published:** 2015-10-09

**Authors:** Rong Ju, Wei Wu, Qiuqin Tang, Di Wu, Yankai Xia, Jie Wu, Xinru Wang

**Affiliations:** 1 Department of Gynaecology and Obstetrics, Nanjing Jiangning Hospital Affiliated to Nanjing Medical University, Nanjing, China; 2 State Key Laboratory of Reproductive Medicine, Institute of Toxicology, School of Public Health, Nanjing Medical University, Nanjing, China; 3 Key Laboratory of Modern Toxicology (Nanjing Medical University), Ministry of Education, Nanjing, China; 4 State Key Laboratory of Reproductive Medicine, Department of Obstetrics, Nanjing Maternity and Child Health Care Hospital Affiliated to Nanjing Medical University, Nanjing, China; 5 State Key Laboratory of Reproductive Medicine, Department of Gynaecology, First Affiliated Hospital of Nanjing Medical University, 300 Guangzhou Road, Nanjing, China; CHA University, REPUBLIC OF KOREA

## Abstract

**Objectives:**

To evaluate whether single nucleotide polymorphisms of *HSD11B1* (rs846908) and *H6PD* (rs6688832 and rs17368528) are associated with polycystic ovary syndrome (PCOS) in Chinese population.

**Materials and Methods:**

A case-control study was implemented to investigate the association between *HSD11B1* and *H6PD* polymorphisms and PCOS. Patients with PCOS (n = 335) and controls (n = 354) were recruited in this study. Genetic variants of *HSD11B1* (rs846908) and *H6PD* (rs6688832 and rs17368528) were analyzed by TaqMan method.

**Results:**

We found a significantly 0.79-fold lower risk of G allele of rs6688832 in control group compared with the patients with PCOS (adjusted OR, 0.79; 95%CI = 0.63–0.99; *P* = 0.040). Additionally, significant difference in the levels of follicle stimulating hormone (FSH) was observed between AA and AG genotype in rs6688832. The rs6688832 AG genotype was associated with lower level of FSH (*P* = 0.039) and higher risk of hyperandrogenism (*P* = 0.016) in patients with PCOS. When all subjects were divided into different subgroups according to age and body mass index (BMI), we found that the frequency of G allele of rs6688832 was significantly higher in controls than that in PCOS patients in the subgroup of BMI > 23 (adjusted OR, 0.70; 95% CI = 0.50–0.98; *P* = 0.037).

**Conclusions:**

Our findings showed a statistical association between *H6PD* rs6688832 and PCOS risk in Chinese population. The G allele of rs6688832 in *H6PD* might exert potential genetic protective role against the development of PCOS, especially in overweight women. PCOS patients with AG genotype of rs6688832 might confer risk to the phenotype of hyperandrogenemia of PCOS.

## Introduction

According to Rotterdam criteria [1], polycystic ovary syndrome (PCOS) is characterized by chronic anovulation and/or oligomenorrhea, clinical and/or biochemical hyperandrogenemia, and polycystic ovary morphology [2]. Studies of familial segregation patterns and twin studies provided convincing evidence for a genetic etiology, but a clear Mendelian inheritance pattern is lacking [3]. It is widely shared that PCOS is the result of a complexity interaction of multiple genetic and environmental factors [4, 5].

Metabolic alterations such as obesity and insulin resistance are frequently accompanied with PCOS [6, 7] and worsen the symptoms of hyperandrogenemia [8–10]. Glucocorticoids (GCs) have huge impact on the regulation of fat distribution, lipid and glucose metabolism. It has been reported that increased peripheral cortisol metabolism was associated with PCOS and independent of body mass index (BMI) [11]. Peripheral GCs metabolism depends on the tissue-specific interconversion of cortisol and cortisone [12–14]. *HSD11B1* and *H6PD* encode 11-beta-hydroxysteroid dehydrogenase type 1 (HSD11B1) and hexose-6-phosphate dehydrogenase (H6PD) and adjust the oxo-reductase activity [15, 16].

*HSD11B1* located at chromosome lq32-41 responsible for the regeneration of glucocorticoids from hormonally-inactive metabolites into active forms in a tissue-specific manner [17]. Many researches have proved that the interconversion of cortisol and cortisone is a primary pathway in glucocorticoids metabolism [17] and demonstrated a pathogenic role for *HSD11B1* in metabolic disease. Polymorphisms in *HSD11B1* have been investigated associated with metabolic phenotype in human, including hyperandrogenemia, type 2 diabetes and hypertension [17–20].

*HSD11B1* oxo-reductase activity requires NADPH (nicotinamide adenine dinucleotide phosphate) provided by *H6PD* [21]. *H6PD* gene located at lp36.2, encodes H6PD as a key enzyme influences 11β-hydroxysteroid dehydrogenase activity in the peripheral metabolism of cortisol [22]. Although previous genetic studies reported a significant association between *H6PD* polymorphisms and the phenotype of PCOS [10, 22, 23], a case-control study in 2011 using DNA samples from 74 patients with PCOS and 31 healthy controls indicated that the *H6PD* gene coding variants rarely (<1.5%) were responsible for hyperandrogenemic PCOS [24].

In this study, we aimed to investigate the polymorphisms rs6688832 and rs17368528 of *H6PD* and rs846908 of *HSD11B1* in an independent population and tried to provide evidence for the association of these polymorphisms with PCOS in Chinese population.

## Materials and Methods

### Study population

All of the subjects in this study were Chinese Han population. A total of 335 PCOS patients and 354 healthy controls were recruited from Jiangsu Province Hospital Affiliated to Nanjing Medical University and Nanjing Maternity and Child Health Care Hospital between March 2005 and February 2009. The PCOS patients (mean age: 28.04 ± 3.87 years) were from those who routinely were referred to our outpatient clinic for PCOS evaluation and selected according to the Rotterdam criteria [1] without a history of fertility and endocrine problems. All subjects were non-smokers. This study protocol was approved by the Institutional Ethics Committee of Nanjing Medical University. Written informed consent was obtained from all participants.

Patients fulfilling any 2 of the following 3 criteria were diagnosed as having PCOS: ligoovulation or oligomenorrhea, clinical or biochemical hyperandrogenemia, and polycystic ovaries morphology [1]. Patients who had diabetes, and congenital adrenal hyperplasia, Cushing syndrome, androgen-secreting tumors, hyperprolactinemia, thyroid disorders, hypertension, or hepatic or renal dysfunction had been excluded from this study. Oligoovulation were determined by menstrual cycle longer than 35 days or less than 21 days with single-phase basal body temperature (BBT) or negative urinary luteinizing hormone. Hyperandrogenemia was determined by the clinical presence of hirsutism (Ferriman-Gallwey score ≥ 6), acne and/or alopecia with high androgen (normal range: total testosterone 0.1–0.75 ng/ml). Polycystic ovaries were defined by ultrasound as the presence of 12 or more small (2–9 mm in diameter) follicles in each ovary and /or increased ovarian volume (> 10 ml; calculated by 0.5 length × width × thickness) [1]. Controls were fertile women (mean age: 30.15 ± 3.08 years), without any clinical or laboratory evidence of PCOS. They were invited for metabolic testing during a routine evaluation. The subgroups of PCOS women and controls were estimated according to age or BMI.

### Sample collection

On the 3rd to the 5th day of a spontaneous menstrual cycle or a progesterone-induced withdrawal bleeding from PCOS patients, heparinized whole-blood samples were gathered in the morning after fasting overnight using tubes containing ethylenediaminetetraacetate (EDTA) (as anticoagulant). Then, every subject had a physical examination including weight, height, waist and hip circumference measurement. The hip circumference was the largest hip size and waist circumference was measured nearby umbilicus when subjects were standing. The plasma of the whole-blood samples was removed and stored at -80°C before analysis. Genomic DNA was extracted from the heparinized venous blood leukocytes of PCOS patients and controls using standard phenol-chloroform method. Follicle stimulating hormone (FSH), luteinizing hormone (LH), prolactin (PRL), testosterone (T), estradiol (E2), dehydroepiandrosterone-sulfate (DHEAS) and fasting insulin (FINS) were detected by chemiluminescence assay. Fasting plasma glucose (FPG) and oral glucose tolerance test (OGTT) were detected by hexokinase-method. The following formulas were adopted to calculate some parameters: HOMA-B% (homeostasis model assessment-B) = 20 × fasting insulin / (fasting plasma glucose-3.5). HOMA-IR (essment-Insulin resistance) = fasting insulin × fasting plasma glucose/22.5 (normal range: < 1.8). QUICKI (quantitative insulin sensitivity check index) = 1 / (logFINS + logFPG (mg/dl)) (normal range: ≥ 0.375). BMI = weight (kg) / height^2^ (m^2^).

### Genotyping

According to the criteria (minimum allele frequency (MAF) ≥ 10% and r^2^ ≥ 0.8), two SNPs rs6688832, rs17368528 in *H6PD* and one SNP rs846908 in *HSD11B1* were selected. Genetic variants of *HSD11B1* and *H6PD* were investigated based on the HapMap database (http://www.hapmap.org). Genotypes were detected by the TaqMan allelic discrimination assay on an ABI 7900HT Fast real-time PCR system (Applied Biosystems). For the quality control, all the genotyping assays were performed without knowing the case/control status and a random 5% of cases and controls were genotyped twice by another technician, yielding a 100% concordant.

### Statistical analysis

Fluorescence data were exported into Excel format and analyzed as scatter points. Hardy-Weinberg equilibrium (HWE) was tested by a goodness-of-fit χ^2^ test. The general clinical and metabolic variables of PCOS patients were expressed as mean ± standard deviation (mean ± SD). The risk of PCOS was estimated as odds ratios (OR) and 95% confidence intervals (95% CI) using unconditional multivariate logistic regression adjusted for age and BMI. All statistical analyses were carried out using Stata (Version 9.0, StataCorp LP, TX, USA), and *P* < 0.05 were considered to be significant.

## Results

The crude genotype frequencies in patients with PCOS (n = 335) and controls (n = 354) are shown in Table 1. The frequencies for patients with PCOS were in HWE expectations (*P* = 0.388 for rs6688832; *P* = 0.408 for rs17368528; *P* = 0.365 for rs846908). We found the genotypic frequencies of the rs6688832 polymorphism had a significant difference in subjects with PCOS compared with controls, with the GG genotype being more commonly found in controls after adjusting for potential covariates (age and BMI) (adjusted OR, 0.62; 95%CI = 0.39–0.99; *P* = 0.045). The GG carriers of polymorphism rs6688832 had 0.62-fold lower risk suffering from PCOS and the G allele of polymorphism rs6688832 was 0.79-fold lower risk (adjusted OR, 0.79; 95%CI = 0.63–0.99; *P* = 0.040). However, no significant difference was found in the genotype frequencies of the polymorphisms *H6PD* rs17368528 and *HSD11B1* rs846908 between patients with PCOS and controls (Table 1).

**Table 1:** Genotype distribution of *H6PD* and *HSD11B1* in women with PCOS and controls.

SNP	Control (n = 354)	Case (n = 335)	*P*	OR (95% CI)	*P* ^a^	OR (95% CI)^a^
**H6PD**						
**rs6688832 (A>G) [n (%)]**						
**AA**	56 (15.82)	72 (21.49)		ref		ref
**AG**	176 (49.72)	175 (52.24)	0.216	0.77 (0.51–1.16)	0.277	0.79 (0.52–1.21)
**GG**	122 (34.46)	88 (26.27)	**0.011**	**0.56 (0.36–0.87)**	**0.045**	**0.62 (0.39–0.99)**
**AG+GG**	298 (84.18)	263 (78.51)	0.056	0.69 (0.47–1.01)	0.112	0.05 (0.48–1.08)
**A**-Allele	288 (40.68)	319 (47.61)		ref		ref
**G**-Allele	420 (59.32)	351 (52.39)	**0.010**	**0.75 (0.61–0.93)**	**0.040**	**0.79 (0.63–0.99)**
**rs17368528 (C>T) [n (%)]**						
**CC**	319 (90.11)	306 (91.34)		ref		ref
**CT**	35 (9.89)	29 (8.66)	0.578	0.86 (0.52–1.45)	0.657	0.89 (0.52–1.51)
**TT**	0 (0)	0 (0)	-	-	-	-
**CT+TT**	35 (9.89)	29 (8.66)	0.578	0.86 (0.52–1.45)	0.657	0.89 (0.52–1.51)
**C**-Allele	673 (95.06)	641 (95.67)		ref		ref
**T**-Allele	35 (4.94)	29 (4.33)	0.588	0.87 (0.53–1.44)	0.665	0.89 (0.53–1.50)
**HSD11B1**						
**rs846908 (G>A) [n (%)]**						
**GG**	189 (53.39)	181 (54.03)		ref		ref
**GA**	140 (39.55)	126 (37.61)	0.700	0.94 (0.69–1.29)	0.930	0.99 (0.71–1.37)
**AA**	25 (7.06)	28 (8.36)	0.595	1.17 (0.66–2.08)	0.686	1.13 (0.62–2.07)
**GA+AA**	165(46.61)	154 (45.97)	0.866	0.97 (0.72–1.32)	0.928	1.01 (0.74–1.39)
**G**-Allele	518 (73.16)	488 (72.84)		ref		ref
**A**-Allele	190 (26.84)	182 (27.16)	0.891	1.02 (0.80–1.29)	0.766	1.04 (0.81–1.33)

^**a**^Adjusted for age and BMI.

OR, odds ratio; CI, confidence interval; Bold values indicate significant findings (*P* < 0.05).

The relationship between the *HSD11B1* and *H6PD* polymorphisms and the clinical parameters in PCOS women are shown in Tables 2 and S1 Table. As shown in Table 2, patients with AG genotype of rs6688832 had a much lower level of FSH than those carrying AA genotype (*P* = 0.039). However, no significant association was found between clinical characteristics and polymorphisms rs846908 in *HSD11B1* and rs17368528 in *H6PD*.

**Table 2:** Clinical characteristics in PCOS women according to different genotypes of *H6PD*.

Parameter	rs6688832					rs17368528				
	AA	AG	GG	*P* ^*a*^	*P* ^*b*^	CC	CT	TT	*P* ^*c*^	*P* ^*d*^
**LH (IU/L)**	12.66 ± 9.87	11.73 ± 6.95	12.36 ± 9.70	0.576	0.881	11.98 ± 8.50	13.31 ± 7.28	-	0.523	-
**FSH (IU/L)**	7.23 ± 1.75	6.56 ±1.77	6.85 ± 1.76	**0.039**	0.287	6.73 ± 1.75	7.27 ± 2.00	-	0.212	-
**T (ng/ml)**	0.80 ± 0.38	0.89 ± 0.38	0.73 ± 0.22	0.178	0.246	0.82 ± 0.34	0.89 ± 0.38	-	0.438	-
**PRL (ng/ml)**	12.89 ± 6.76	15.95 ± 20.19	13.90 ± 7.93	0.196	0.536	14.53 ± 16.06	17.17 ± 8.56	-	0.298	-
**E**_**2**_ **(pg/ml)**	54.51 ± 46.09	62.08 ± 43.36	55.13 ± 43.27	0.363	0.948	58.65 ± 44.11	57.08 ± 42.65	-	0.889	-
**Fasting glucose (mmol/L)**	5.28 ± 0.90	5.87 ± 4.64	5.91 ± 2.39	0.353	0.202	5.79 ± 3.79	5.28 ± 0.70	-	0.336	-
**Fasting insulin (μIU/ml)**	13.58 ± 10.01	13.30 ± 10.20	9.76 ± 5.72	0.901	0.076	12.22 ± 9.35	13.87 ± 8.10	-	0.543	-
**DHEAS (μmol/L)**	7.08 ± 2.45	7.94 ± 3.04	8.99 ± 2.91	0.419	0.113	8.01 ± 2.90	7.66 ± 3.37	-	0.844	-
**HOMA-IR**	3.42 ± 2.83	4.66 ± 9.79	3.37 ± 3.72	0.393	0.958	4.12 ± 7.82	3.54 ± 2.49	-	0.597	-
**HOMA-B%**	151.79 ± 102.92	163.20 ± 112.27	1106.87 ± 5135.34	0.676	0.343	433.12 ± 2765.62	157.20 ± 80.03	-	0.337	-
**Fasting glucose-insulin ratio**	12.77 ± 11.17	9.88 ± 6.18	11.24 ± 5.95	0.242	0.552	11.13 ± 7.84	8.78 ± 4.31	-	0.127	-
**QUICKI (mg/dl)**	0.34 ± 0.05	0.33 ± 0.04	0.33 ± 0.03	0.451	0.626	0.34 ± 0.04	0.33 ± 0.03	-	0.480	-
**LH/FSH**	1.70 ± 1.16	1.94 ± 1.73	1.87 ± 1.60	0.345	0.553	1.87 ± 1.64	1.82 ± 0.93	-	0.851	-

Values are mean ± SD.

^a^ *P* values between AA and AG genotypes of rs6688832.

^b^ *P* values between AA and GG genotypes of rs6688832.

^c^ *P* values between CC and CT genotypes of rs17368528.

^d^ *P* values between CC and TT genotypes of rs17368528.

In order to investigate the stratification between rs6688832 and the risk of PCOS, we analyzed the different frequencies between the A and G allele of rs6688832 in both patients with PCOS and controls (Table 3). In Table 1, we found the G allele frequency of polymorphism rs6688832 was significantly lower in the patients with PCOS than controls (*P* = 0.040). When we divided subjects into subgroups according to age and BMI, we observed that the frequency of rs6688832 G allele was significantly higher in controls than that in PCOS patients in the subgroup of BMI >23 (adjusted OR, 0.70; 95% CI = 0.50–0.98; *P* = 0.037). However, no significant difference was found between the G allele and A allele frequencies of rs6688832 in the subgroups divided by age (Table 3).

**Table 3:** Association and stratification analysis between rs6688832 and risk of PCOS.

Variables	N (control/case)	Alleles (control/case)				*P* ^a^	OR (95% CI)^a^
		A-Allele		G-Allele			
		n	%	n	%		
**Total**	708/670	288/319	40.68/47.61	420/351	59.32/52.39	**0.040**	**0.79 (0.63–0.99)**
**Age (years)**							
≤ 30	358/466	147/222	41.06/47.64	211/244	58.94/52.36	0.082	0.78 (0.59–1.03)
> 30	350/204	141/97	40.29/47.55	209/107	59.71/52.45	0.122	0.76 (0.53–1.08)
**BMI**							
≤ 23	390/358	158/165	40.51/46.09	232/193	59.49/53.91	0.224	0.83 (0.62–1.12)
> 23	318/312	130/154	40.88/49.36	188/158	59.12/50.64	**0.037**	**0.70 (0.50–0.98)**

OR, odds ratio; CI, confidence interval. Bold values indicate significant findings (*P* < 0.05).

^**a**^Adjusted for age and BMI, where it was appropriate.

To analyze the relationship between the three polymorphisms and hyperandrogenemia, we divided patients with PCOS into two subgroups: hyperandrogenemia and without hyperandrogenemia (Table 4). In hyperandrogenemia group, patients with AG genotype of rs6688832 had a much higher frequency and had a statistical difference compared with AA genotypes (adjusted OR, 2.36; 95% CI = 1.14–4.89; *P* = 0.021). No significant difference was found in genotype frequencies distributions of the polymorphisms rs17368528 of *H6PD* and rs846908 of *HSD11B1* (Table 4).

**Table 4:** Distribution of four SNPs in PCOS patients with hyperandrogenemia and without hyperandrogenemia.

SNP	Without hyperandrogenism	Hyperandrogenism	*P*	OR (95%CI)	*P* ^a^	OR (95%CI)^a^
**H6PD**						
r**s6688832 (A>G) [n (%)]**						
**AA**	23 (26.74)	22 (18.49)		ref		ref
**AG**	32 (37.21)	74 (62.18)	**0.016**	2.42 (1.18–4.95)	**0.021**	2.36 (1.14–4.89)
**GG**	31 (36.05)	23 (19.33)	0.531	0.78 (0.35–1.72)	0.622	0.81 (0.92–1.14)
**AG+GG**	63 (73.26)	97 (81.51)	0.161	1.61 (0.83–3.13)	0.176	1.60 (0.81–3.17)
**A**-Allele	78 (45.35)	118 (49.58)		ref		ref
**G**-Allele	93 (54.65)	120 (50.42)	0.398	0.84 (0.57–1.25)	0.457	0.86 (0.57–1.28)
**rs17368528 (C>T) [n (%)]**						
**CC**	80 (93.02)	106 (89.08)		ref		ref
**CT**	6 (6.98)	13 (10.92)	0.340	1.64 (0.60–4.49)	0.455	1.48 (0.53–4.15)
**TT**	0 (0)	0 (0)	-	-	-	-
**CT+TT**	6 (6.98)	13 (10.92)	0.340	1.64 (0.60–4.49)	0.455	1.48 (0.53–4.15)
**C**-Allele	166 (96.51)	225 (94.54)		ref		ref
**T**-Allele	6 (3.49)	13 (5.46)	0.352	1.60 (0.60–4.29)	0.464	1.46 (0.53–3.97)
**rs846908 (G>A) [n (%)]**						
**GG**	55 (58.51)	80 (72.07)		ref		ref
**GA**	34 (36.17)	27 (24.32)	0.346	0.75 (0.42–1.35)	0.311	0.73 (0.40–1.34)
**AA**	5 (5.32)	4 (3.61)	0.266	0.54 (0.18–1.60)	0.437	0.63 (0.20–2.01)
**GA+AA**	39 (41.49)	31 (27.93)	0.240	0.72 (0.41–1.25)	0.249	0.71 (0.40–1.27)
**G**-Allele	144 (76.60)	187 (84.23)		ref		ref
**A**-Allele	44 (23.40)	35(15.77)	0.187	0.74 (0.48–1.15)	0.236	0.76 (0.48–1.20)
**SNP**	**Without hyperandrogenism**	**Hyperandrogenism**	***P***	**OR (95% CI)**	***P*** ^**a**^	**OR (95% CI)** ^**a**^
**H6PD**						
r**s6688832 (A>G) [n (%)]**						
**AA**	23 (26.74)	22 (18.49)		ref		ref
**AG**	32 (37.21)	74 (62.18)	**0.016**	2.42 (1.18–4.95)	**0.021**	2.36 (1.14–4.89)
**GG**	31 (36.05)	23 (19.33)	0.531	0.78 (0.35–1.72)	0.622	0.81 (0.92–1.14)
**AG+GG**	63 (73.26)	97 (81.51)	0.161	1.61 (0.83–3.13)	0.176	1.60 (0.81–3.17)
**A**-Allele	78 (45.35)	118 (49.58)		ref		ref
**G**-Allele	93 (54.65)	120 (50.42)	0.398	0.84 (0.57–1.25)	0.457	0.86 (0.57–1.28)
**rs17368528 (C>T) [n (%)]**						
**CC**	80 (93.02)	106 (89.08)		ref		ref
**CT**	6 (6.98)	13 (10.92)	0.340	1.64 (0.60–4.49)	0.455	1.48 (0.53–4.15)
**TT**	0 (0)	0 (0)	-	-	-	-
**CT+TT**	6 (6.98)	13 (10.92)	0.340	1.64 (0.60–4.49)	0.455	1.48 (0.53–4.15)
**C**-Allele	166 (96.51)	225 (94.54)		ref		ref
**T**-Allele	6 (3.49)	13 (5.46)	0.352	1.60 (0.60–4.29)	0.464	1.46 (0.53–3.97)
**rs846908 (G>A) [n (%)]**						
**GG**	55 (58.51)	80 (72.07)		ref		ref
**GA**	34 (36.17)	27 (24.32)	0.346	0.75 (0.42–1.35)	0.311	0.73 (0.40–1.34)
**AA**	5 (5.32)	4 (3.61)	0.266	0.54 (0.18–1.60)	0.437	0.63 (0.20–2.01)
**GA+AA**	39 (41.49)	31 (27.93)	0.240	0.72 (0.41–1.25)	0.249	0.71 (0.40–1.27)
**G**-Allele	144 (76.60)	187 (84.23)		ref		ref
**A**-Allele	44 (23.40)	35(15.77)	0.187	0.74 (0.48–1.15)	0.236	0.76 (0.48–1.20)

Values are mean ± SD.

^a^Adjusted for age and BMI.

## Discussion

It is universally acknowledged that PCOS is a kind of complex heterogeneous diseases [4,5], and the etiology of the disease is still unknown. The phenotypes of PCOS endocrine disorder include hyperandrogenemia, high LH / FSH (luteinizing hormone /folliclestimulating hormone) ratio, irregular menstruation, obesity, insulin resistance and/or infertility. Previous study has demonstrated that metabolic abnormalities are associated with PCOS [8,9]. Many researches proved that the *HSD11B1* linked to various metabolic abnormalities and the importance has been testified in obesity and insulin resistance and other components of the metabolic syndrome [19, 21, 25, 26]. *H6PD* gene has been reported associated with PCOS and might influence its phenotype by influencing adrenal activity [10,24]. Therefore, *H6PD* and *HSD11B1* can be considered as key genes to explain the metabolic abnormalities of PCOS.

Polymorphisms are common DNA sequence variations among population playing an important role in the development of several hereditary diseases. As key genes of cortisol metabolism, *HSD11B1* and *H6PD* play an important role in human endocrine regulation. In our study, we observed a significantly higher frequency of rs6688832 GG genotype and G allele in the controls compared with patients with PCOS. No significant different distribution of genotypes or allele of polymorphism rs17368528 and rs846908 were observed between patients with PCOS and controls. After dividing both patients and controls into two subgroups based on their age or BMI, we found G allele of rs6688832 could protect overweight women from consequence of PCOS. In order to explore the probable mechanisms, we performed secondary structure prediction of the H6PD mRNA sequence using the algorithm RNAfold [27]. As shown in S1 Fig, black arrowheads pointed the significant changes of RNA structure both under minimum free energy (MFE) secondary structure and centroid secondary structure. The changes in RNA structure suggested that the rs6688832 polymorphism might affect the stability of the RNA or the interaction of the RNA with other macromolecules. Together, our result indicate that the G allele of rs6688832 in *H6PD* exert a potential genetic protective character against the development of PCOS.

In addition, we examined the association between the three polymorphisms of *HSD11B1* and *H6PD* and the clinical features of PCOS, we found a significant difference in FSH level between AA and AG genotypes of rs6688832 in *H6PD*. Compared with AA genotype, we found the PCOS patients with AG genotype had a lower level of FSH. No significant difference was found between clinical parameters and rs17368528 in *H6PD* and rs846908 in *HSD11B1*. Then we divided patients into two groups by testosterone level to evaluate the distribution of the three genotypes, we also found a significant difference between AA and AG genotype of rs6688832 and the AG genotype was associated with hyperandrogenism, but no statistical different distribution of polymorphism rs17368528 of *H6PD* and rs846908 of *HSD11B1* were observed. This consistency might be contributed to several reasons. Firstly, it might be related to the dysfunction of hypothalamus pituitary ovary axis due to the hyperandrogenism. Secondly, FSH was inhibited in a low level in patients with PCOS due to the high level of estrogen converted by the androgen. Thirdly, FSH can activate the enolization enzyme, promote the androgen into estrogen and induce the formation of LH receptor. The lower level of FSH can decrease the activity of enolization enzyme, inhibit the conversion of androgen into estrogen and increase the level of androgen. Our study indicated that the AG genotype of rs6688832 was associated with the phenotype of hyperandrogenism of PCOS. A previous genetic study reported that *H6PD* variants were associated with the PCOS phenotype by influencing obesity, insulin resistance and rarely responsible for hyperandrogenism [24]. In this study, we found the variants of rs6688832 are associated with the phenotype of hyperandrogenism of PCOS. The inconsistency, on one hand, might be due to different genetic backgrounds and environmental factors which cause differences in association studies. On the other hand, the sample size in our study is larger and is more likely to find the real association.

In summary, our results showed that the GG genotype and G allele of rs6688832 in *H6PD* might act as a genetic protective role against the development of PCOS. Patients with PCOS carrying AG genotype of rs6688832 could influence FSH level and associated with the phenotype of hyperandrogenism of PCOS. Although it is not known at present as to how polymorphisms in the *H6PD* lead to different phenotype of PCOS, it is hypothesized that it might play a certain role and affect the function of the hypothalomo-pituitary-gonadal axis in humans. Our findings still need to further confirmed by larger sample size studies with a different ethnicity in the occurrence of hyperandrogenemia in patients with PCOS. Further studies are required to find out the molecular mechanism of the rs6688832 AG genotype on the risk of hyperandrogenemia and PCOS.

## Supporting Information

S1 Fig Prediction of the secondary structure of mRNA sequence containing the rs6688832 variants. A and B represent MFE (minimum free energy) secondary structure and centroid secondary structure respectively. (a) and (b) represent A allele and G allele respectively. Black arrowheads mean the significant changes of RNA structure. All structures were predicted using the algorithm RNAfold.
(TIF)

S1 Table Clinical characteristics in PCOS women according to different genotypes of rs846908 in *HPD11B1*. (DOC)
