# Supplementary figures and images for: Association Analysis between the Polymorphisms of HSD11B1 and H6PD and Risk of Polycystic Ovary Syndrome in Chinese Population
Source: PLoS One. 2015 Oct 9;10(10):e0140326. doi: 10.1371/journal.pone.0140326 (PMC4599835; doi:10.1371/journal.pone.0140326)

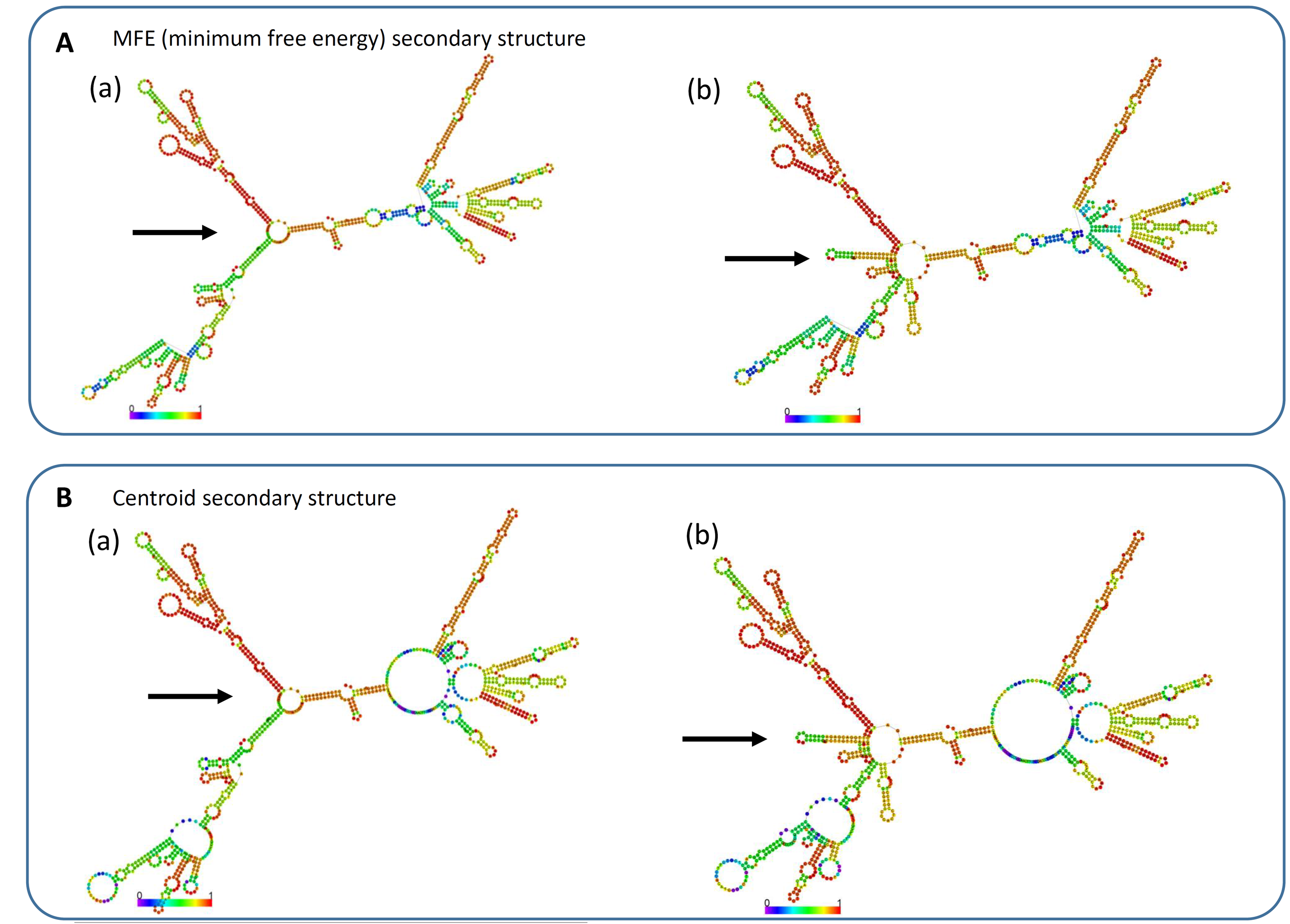

Supplement: S1 Fig — A and B represent MFE (minimum free energy) secondary structure and centroid secondary structure respectively. (a) and (b) represent A allele and G allele respectively. Black arrowheads mean the significant changes of RNA structure. All structures were predicted using the algorithm RNAfold. (TIF) [file pone.0140326.s001.tif]
