# Supplementary material for: Association Analysis between the Polymorphisms of HSD11B1 and H6PD and Risk of Polycystic Ovary Syndrome in Chinese Population
Source: PLoS One. 2015 Oct 9;10(10):e0140326. doi: 10.1371/journal.pone.0140326 (PMC4599835; doi:10.1371/journal.pone.0140326)
Supplement: S1 Table — (DOC) [file pone.0140326.s002.doc]

S1 Table. Clinical characteristics in PCOS women according to different genotypes of rs846908 in *HPD11B1*.

| **Parameter** | rs846908 | | | | |
| --- | --- | --- | --- | --- | --- |
|  | **GG** | **GA** | **AA** | ***P^a^*** | ***P^b^*** |
| **LH (IU/L)** | 11.44 ± 7.44 | 12.67 ± 8.96 | 13.98 ± 11.71 | 0.308 | 0.440 |
| **FSH (IU/L)** | 6.72 ± 1.69 | 6.73 ±1.86 | 7.51 ± 1.86 | 0.976 | 0.107 |
| **T (ng/ml)** | 0.82 ± 0.33 | 0.85 ± 0.38 | 0.80 ± 0.33 | 0.603 | 0.770 |
| **PRL (ng/ml)** | 15.45 ± 19.99 | 13.53 ± 7.80 | 16.36 ± 8.22 | 0.395 | 0.769 |
| **E_2_ (pg/ml)** | 58.13 ± 45.36 | 58.59 ± 42.03 | 60.69 ± 45.57 | 0.946 | 0.844 |
| **Fasting glucose (mmol/L)** | 5.92 ± 4.68 | 5.66 ± 1.91 | 4.85 ± 0.26 | 0.707 | 0.091 |
| **Fasting insulin (µIU/ml)** | 12.90 ± 10.42 | 12.33 ± 8.00 | 8.17 ± 5.26 | 0.737 | 0.242 |
| **DHEAS (µmol/L)** | 8.28 ± 3.06 | 7.17 ± 2.32 | 10.79 ± 3.57 | 0.196 | 0.200 |
| **HOMA-IR** | 4.49 ± 9.82 | 3.82 ± 3.65 | 1.85 ± 1.23 | 0.643 | 0.069 |
| **HOMA-B%** | 655.26 ± 3661.94 | 148.90 ± 98.15 | 129.16 ± 82.30 | 0.319 | 0.302 |
| **Fasting glucose-insulin ratio** | 10.80 ± 8.44 | 10.29 ± 5.68 | 15.96 ± 10.63 | 0.715 | 0.171 |
| **QUICKI (mg/dl)** | 0.34 ± 0.04 | 0.33 ± 0.03 | 0.36 ± 0.04 | 0.574 | 0.141 |
| **LH/FSH** | 1.82 ± 1.70 | 1.95 ± 1.50 | 1.79 ± 1.17 | 0.576 | 0.962 |
